# A bibliometric search of citation classics in anesthesiology

**DOI:** 10.1186/1471-2253-11-24

**Published:** 2011-12-12

**Authors:** Ravi S Tripathi, James M Blum, Thomas J Papadimos, Andrew L Rosenberg

**Affiliations:** 1 University of Michigan Medical School, Department of Anesthesiology, 1H247 UH, SPC 5048, 1500 East Medical Center Drive, Ann Arbor, Michigan 48109-5048, USA; 2 The Ohio State University Medical Center, Department of Anesthesiology, 410 West 10th Avenue, Columbus, Ohio 43210, USA; 3 University of Michigan Medical School, Department of Anesthesiology, 1H247 UH, SPC 5048, 1500 East Medical Center Drive, Ann Arbor, Michigan, 48109-5048, USA; 4 The Ohio State University Medical Center, Department of Anesthesiology, 410 West 10th Avenue, Columbus, Ohio, 43210, USA

## Abstract

**Background:**

Articles cited counts are catalogued and help identify landmark papers. This study provides a citation classics of anesthesiology literature using the framework of subspecialties to provide a review of well-developed areas of research in anesthesiology.

**Methods:**

A comprehensive list of the most-cited articles in anesthesia was compiled using a bibliometric database and general search terms such as "anesthesia" as well as subspecialty-specific search terms. Queries were reviewed for relevance to anesthesiology practice, categorized by subspecialty, and ranked according to their citation counts.

**Results:**

The database resulted in 2519 articles published between 1945 and 2008. The specialty areas most represented were chronic pain medicine (11%), pharmacology (9%), and pain sciences (9%).

**Conclusions:**

This citations classic allows for advances in anesthesiology and its subspecialties to be highlighted as well to provide useful manuscripts to guide patient care, direct future research, and serve as sources for future academic pursuit.

## Background

It is ironic that as electronic access to medical literature becomes more pervasive, the ability for an individual to maintain a semblance of broad awareness of that body of knowledge becomes more difficult. The diversity of diseases, the patients, and the basic sciences that encompass the specialty of anesthesiology and its related specialties is reflected by a similar heterogeneity of the journals in which anesthesiology knowledge is published. As this body of knowledge increases, it is important to enhance methodologies that identify especially relevant and important papers within the overall field, as well as within its multiple subspecialties. With the development of Internet-based search engines, numerous methods to search for relevant medical literature now exist. While these databases are easy to use, the results of basic keyword or topic searches are often overwhelming and shed little light on the most relevant articles. There is a need to improve a practioners' ability to quickly find important articles.

Articles that have value to others are often cited in subsequent manuscripts. These referenced papers are catalogued in bibliometric resources that track the number of times a paper is cited. Because the vast majority of published articles are never referenced even once, those that are cited often arguably had significant influence. The more times an article is cited, the more likely it is to have impacted the field and patient care [1]. These collections of cited articles are sometimes referred to as 'Citation Classics.' It is argued that citation classics have their limitations and the enthusiasm for defining manuscripts as such is varied [2-5]. However, citation classics are considered a reasonable proxy for the focus of contemporary experts in a field at a given period, reflect the state of scientific inquiry, and have been shown to follow proposed hierarchy of evidence with meta-analysis being the most-often cited articles and case reports the least cited [6].

In 1987, Garfield catalogued citation classics from the *Journal of the American Medical Association *[7]. Since then, similar studies have been performed within multiple medical specialties [1,8-16]. These reviews have used different approaches for a variety of medical specialties, and investigations focusing on frequently-cited literature within anesthesiology have received little attention [17-21]. The most recent of these publications is now almost seven years old and does not include any articles published after 1997 [21]. Among the limitations of these articles are the authors' confining their search only to anesthesiology journals [21], or in the case of Hall et al., limiting their search to a single anesthesiology journal [17]. Another limitation of previous citation classic surveys has been the paucity of attempts to explore for anesthesiology-centric articles within medical specialty areas not exclusive to anesthesiology (example: pain, pediatrics, obstetrics, neurosciences). Additionally, anesthesiology subspecialties have received essentially no careful review, and there are no data to date regarding the subspecialty influence, as reflected by citations counts for these areas [22].

The primary aim of this study was to expand on these earlier works by examining literature related to the field of anesthesiology in both anesthesiology and non-anesthesiology journals. Similar to previous citation classics [16], databases were searched not only by specific journals, but also by specific search terms such as anesthesiology.

The second goal of this paper was to improve the capture of highly-cited articles, with a primary subject pertaining to anesthesiology issues within subspecialties that may have been previously missed in surveys that did not explicitly make this focus a priority.

## Methods

To develop the most comprehensive list of cited articles in anesthesiology and anesthesiology-related subspecialties, the search strategy was conducted using three methods. All of these searches used the ISI Web of Knowledge databases (http://www.isiwebofknowledge.com, Thomson Rheuters, 2008). Two of ISI Web of Knowledge's databases are the Journal Citation Report (JCR) and Science Citation Expanded (SCI Expanded). JCR is a resource that lists the names of over 5900 journals, both scientific and technical, for their bibliometric information and impact factors. SCI Expanded is an index of articles in over 6670 scientific journals that include bibliographic information, cited references, and citation counts. All queries were performed during January and February 2010. The SCI was limited to articles published until and including 2009.

### Journal Search

Similar to previous citation classic for anesthesiology [21], the search began by identifying journals with the subject category "anesthesiology" using JCR 2008. Of the 22 journals designated as anesthesiology, individual queries were performed for each of 19 journals published in the English language (Table 1). SCI Expanded was then searched for articles published within these journals. Bibliometric data on articles that were cited at least 100 times were collected, similar to previous citation investigations [16].

**Table 1:** Anesthesiology journals used for journal search.

Acta Anaesthesiologica Scandinavica
Anaesthesia and Intensive Care
Anaesthesia
Anaesthesist
Anesthesia &Analgesia
Anesthesiology
British Journal of Anaesthesia
Canadian Journal of Anaesthesia
Clinical Journal of Pain
European Journal of Anesthesiology
European Journal of Pain
International Journal of Obstetric Anesthesia
Journal of Cardiothoracic and Vascular Anesthesia
Journal of Clinical Anesthesia
Journal of Neurosurgical Anesthesiology
Minerva Anesthesiologica
Pain
Pediatric Anesthesia
Regional Anesthesia and Pain Medicine

### Keyword Search

The second strategy was used to create a more complete and comprehensive list and to capture relevant articles not within anesthesiology journals. SCI Expanded was queried by keyword. To find articles related to anesthesiology among all scientific journals, the search terms "anesthes*" and "anaesth*" were used to retrieve articles that contained the keywords anesthesia, anesthesiology, anesthesiologist, anesthetists, anesthetics, anaesthesia, or anaesthesist. The symbol "*" is a wildcard to retrieve all search items that start with the preceding text. As done with the search by journal, bibliometric information on articles that had been cited more than 100 times in our database and published in English language were collected.

### Subspecialty Search

To organize the retrieved articles, this study defined 14 areas of anesthesiology practices that included the breadth of the field. Due to previous publication in the area of critical care [16], critical care was omitted from this study. SCI Expanded was searched by terms within these subject areas (see Table 2). This strategy resulted in 65 separated queries, from which duplicate articles were removed. Articles with at least 100 citations and published in the English languate were used for this analysis.

**Table 2:** Specific anesthesiology subspecialties areas and search terms used.

Subject Area	Focus	Search Terms
Airway management	Intubation, difficult airway	"difficult-airway" intubation
Cardiothoracic and vascular anesthesiology	Cardiothoracic anesthesiology and intraoperative cardiac pathology	"bypass" "cardiac" "cardio*" "heart" "transfusion" "thoracic" "single lung" "one lung" "lung isolation" "vascular" "aneurysm"
General anesthesiology and physiology	Articles that pertained to field as a whole	"sedation" "monitored-anesthesia-care" "MAC" "line-placement" "cannula*"
Head and neck surgery (including neurophysiology)	Neuroanesthesia and anesthesiology for head and neck surgery	"neurosurgery" "crani*" "cerebral-blood-flow" "intracranial-pressure" "carotid endart*"
Monitors	Hemodynamic monitors and monitors of depth of anesthesiology (excluding monitors of surgical techniques)	"monitor" "safety" "record" "inform*" "echo*"
Obstetric anesthesiology	Including environmental and occupational studies	"obstetric anes*" "labor analgesia"
Pain (basic science and clinical management)	Basic sciences included pharmacology and acute physiology, while chronic included clinical management of patients with chronic pain	"pain" "opioid" "opiate"
Pediatric anesthesiology	Including both anesthesiology and pain	"pediatric" "paediatric"
Preoperative medicine	Precardiovascular screening for non-cardiac surgery, preoperative optimization, and other disease-risk stratification	"preop*" "periop*" "intraop*"
Postoperative care	Postoperative pain management, nausea and vomiting, and other physiologic complications of anesthesiology (excluding pulmonary)	"postop*" "PACU" "postanesthesia" "postanaesthesia"
Pharmacology	Mainly volatile anesthetics, intravenous anesthetics, and neuromuscular blocking drugs (local anesthetics and opiods were addressed elsewhere)	"local anes" "local anaes" "nondepolariz*" "succinylcholine" "malignant hyper*" "intravenous anes" "intravenous anaes" "inhalation anes*" "volatile anes*" "neuromuscular-block*" "paralytic"
Regional and neuraxial anesthesiology	Safety and use of regional and neuraxial anesthesiology including pharmacology and physiology	"regional anes*" "regional anaes*" "neuraxial anes*" "neuraxial anaes*" "epidural" "spinal" "subarachnoid" "intrathecal" "ambulatory surgery"
Pulmonary	Intraoperative ventilatory management, pulmonary physiology, and postoperative pathology	"ventilator" "hypoxia"
Fluid management	Intraoperative fluid optimization and transfusion medicine	"transfusion" "fluid" "blood"

* Indicates a wildcard to return any string of characters

"Subject" indicates the subspecialty area. "Focus" indicates the desired content of the subject or subspecialty area. "Search terms" are the actualy keywords used for the searches.

The authors evaluated each article to ensure its relevance to anesthesiology by reviewing citation information available, including article title, source journal, keywords, and abstract. Criterion included articles that were clinically relevant to the practice of anesthesiology. For example, articles regarding the preoperative diagnosis of carotid stenosis and the decision to treat medically or operatively were eliminated. Similarly, articles focusing on the postoperative surgical complications were also eliminated. On the other hand, articles that focused on the intraoperative management of patients undergoing carotid endartectomy were included. Postoperative complications and care that were related to anesthetic practice were kept. Articles that were no longer clinically relevant were removed. For example, due to the questionable future of aprotinin, only key articles regarding the use of this drug are highlighted. This study's preliminary survey and previous studies [20] have shown that anesthesiology is dominated by pain literature; thus, this subspecialty was divided into two categories: acute/basic pain science and chronic pain management. Additional articles with a primary focus of basic science/mechanism, animal studies, and research methodology were excluded since the aim of this paper was to provide the practicing anesthetists with clinically-relevant articles.

Once the irrelevant articles were removed, the authors categorized the articles according to subspecialty. Each of these categories was reviewed with leaders in their respective field at the University of Michigan to ensure validity of the searches. Articles are presented according to their subspecialty in descending order according to their citation counts. For articles published simultaneously in more than one journal, the cumulative citation count is reflected. The 20 most-cited articles in each subspecialty are presented.

## Results

Initial search strategies resulted in 19,478 articles. After excluding duplicates and irrelevant articles in the manner listed in methods, the database of articles specific to the conduct of anesthesiology itself included only 2519 articles (13% of the original search). The articles were published between 1945 (Whitacre's "Clinical observation on the use of curare in anesthesia," Anesthesiology) and 2008 (Devereaux's POISE Trial, Lancet). The majority of the most-cited publications occurred between 1980 and 1990. Of the 10 most-cited articles, the mean publication year was 1981.

The articles came from 103 distinct journals. The journals with the most articles were *Anesthesiology *(27%, n = 673), Pain (22%, n = 563), and *British Journal of Anaesthesia *(8%, n = 202). Seventy-two percent of the articles (n = 1,804) were published in anesthesiology-related journals as identified by JCR 2008 (see Table 1). The "non-anesthesiology" journal with the most highly-cited anesthesiology-related articles was the *Lancet *(2%, n = 42).

Of the 1250 categorized articles, the most common topics were chronic pain medicine (11%, n = 139), pharmacology (9%, n = 109), and acute and basic pain sciences (9%, n = 108).

Pharmacology (n = 6, 38%) was the most-cited category prior to and during the 1960s, with landmark papers such as Egler's discussion of minimum alveolar concentration in *Anesthesiology *(1965). After 1970, pain articles predominate with landmark papers such as Melzack's "The Mcgill Pain Questionnaire" in Pain (1975). This prevalence of pain articles continues for the remainder of the citations chronologically.

Articles that are more-recently published will have a shorter exposure to the medical community; arguably, their times cited may be less often than older papers that have a longer presence in the literature. In Table 3 we have presented the articles with the highest citation count for each year for the previous 20 years. This was done to highlight articles that have likely influenced clinical care but have not reached their citation peak due to their infancy, and thus, are not presented in our citation classics by subspecialty. The 20 most-cited articles by specialty are presented in Additional file 1 with their time cited and their overall rank in the entire database designated.

**Table 3:** 20 Years of Most cited articles by year since 1989.

Year	First Author. Title. Journal. (# Rank in Overall Database/Times Cited)
1989	Bidstrup, BP. Reduction in blood-loss and blood use after cardiopulmonary bypass with high-dose aprotinin (trasylol). J Thorac Cardiov Sur 1989. (#95/398)
1990	Paulson, OB. Cerebral autoregulation. Cerebrovas Brain Met 1990. (#25/697)
1991	Woolf, CJ. The induction and maintenance of central sensitization is dependent on N-methyl-D-aspartic acid receptor activation - implications for the treatment of postinjury pain hypersensitivity states. Pain 1991. (#9/1059)
1992	Watcha, MF. Postoperative nausea and vomiting - its etiology, treatment, and prevention. Anesthesiology 1992. (#22/713)
1993	Coderre, TJ. Contribution of central neuroplasticity to pathological pain - review of clinical and experimental-evidence. Pain 1993. (#7/1183)
1994	Vandermeulen, EP. Anticoagulants and spinal epidural-anesthesia. Anesth Analg 1994. (#118/365)
1995	Vlaeyen, JWS. Fear of movement (re)injury in chronic low-back-pain and its relation to behavioral performance. Pain 1995. (#67/445)
1996	Roach, GW. Adverse cerebral outcomes after coronary bypass surgery. New Engl J Med 1996. (#15/843)
1997	Mihic, SJ. Sites of alcohol and volatile anaesthetic action on gaba(a) and glycine receptors. Nature 1997. (#29/670)
1998	Rampil, IJ. A primer for eeg signal processing in anesthesia. Anesthesiology 1998. (#70/443)
1999	Sindrup, SH. Efficacy of pharmacological treatments of neuropathic pain: an update and effect related to mechanism of drug action. Pain 1999. (#45/545)
2000	Vlaeyen, JWS. Fear-avoidance and its consequences in chronic musculoskeletal pain: a state of the art. Pain 2000. (#36/632)
2001	Farrar JT. Clinical importance of changes in chronic pain intensity measured on an 11-point numerical pain rating scale. Pain. (#40/578)
2002	Eagle KA. ACC/AHA guidelines update for perioperative cardiovascular evaluation for noncardiac surgery. Circulation. (#79/433)
2003	Sandham JD. A randomized, controlled trial of the use of pulmonary-artery catheters in high-risk surgical patients. New Engl J Med. (#131/348)
2004	Myles PS. Bispectral index monitoring to prevent awareness during anaesthesia. Lancet. (#366/214)
2005	Apkarian AV. Human brain mechanisms of pain perception and regulation in health and disease. Eur J Pain. (#219/282)
2006	Mangano DT. The risk associated with aprotinin in cardiac surgery. New Engl J Med. (#129/350)
2007	Dworkin RH. Pharmacologic management of neuropathic pain. Pain. (#1105/139)
2008	Devereux PJ. Effects of extended-release metoprolol succinate in patients undergoing non-cardiac surgery (POISE trial). Lancet. (#991/147)

## Discussion

Access to the world's contemporary scientific literature is increasingly more available via medical libraries, Internet data repositories, and web-based search engines. However, without preexisting knowledge of the most influential articles, finding the most relevant articles is difficult. The purpose of this study was to provide updated citation classics of anesthesiology literature using the framework of subspecialties within the general field to provide a review of well-developed areas of research in anesthesiology. As the field of anesthesiology advances in research and clinical science, this review would assist anesthesiology providers with areas of anesthesiology that are already well studied as well as shows areas with a paucity of research to guide short- and long-term goals for departments, divisions, and collaborations.

Unlike a previous study for critical care articles [16], this study found the majority of highly-cited anesthesiology articles within primarily anesthesiology journals. In this study of anesthesiology citation classics, 72% of the articles came from publications that were designated as anesthesiology journals by the JCR. This is likely to reflect either the paucity of anesthesiology studies being submitted to non-anesthesiology journals, or a reflection of a quality or interest gap for anesthesiology-related studies in non-anesthesiology journals. As the practice of anesthetists expands from the operating room to the perioperative arena--including preoperative outpatient clearance, perioperative pain management, postoperative intensive care management, and even areas of palliative care--anesthesiology-related articles should show more prevalence in non-anesthesiology and general medical journals. Departmental chairs, administrators, and committees could use the placement of academic work in non-anesthesiology journals as a marker of excellence and significant contribution to the medical community by their faculty and divisions.

The keyword "anesthesia" is surprisingly not effective in retrieving anesthesiology-focused articles. To begin, the majority of these manuscripts are related to surgical procedures, outcomes, and complications with no focus on the conduct or issues related to anesthesiology. Furthermore, the specificity for "anesthesia" as a search term is also quite poor. For example, when "anesthes*" is queried in SCI Expanded to retrieve articles containing either anesthesia or anesthesiology, one of the most-cited articles retrieved is "Multilineage cell from human adipose tissue: implications for cell-based therapy" by Zuk and colleagues in *Tissue Engineering *(2001) due to the use of the phrase "under local anesthesia" in their abstract; however, this article is clearly not relevant to the practicing anesthetists. Another explanation for the poor specificity for keywords may relate to the previous finding that authors list various keywords in their manuscripts in order to increase the number of times their articles are referenced. It has been suggested that authors carefully choose the words used in their abstract to improve the chances of their article being found and cited [23]. Also, during a pilot search by keyword "anesthesia," many classics articles were not included. One explanation includes articles published before the mid-1980s did not list keywords. Therefore, these landmark studies were less likely to be identified by the search "anesthesia."

A somewhat unique aspect of this recent survey of anesthesiology citation classics is the unique focus on anesthesiologist specialties, a method not previously performed. Examining the individual subspecialties, the predominance of pain articles and their high-citation rate is not surprising. Pain is a clinical entity that is used by many fields in medicine--anesthesiologist, physical medicine and rehabilitation, surgery, medicine, psychiatry and addiction medicine, nursing--so these articles have interest to a larger audience than articles on the basics of anesthesiologist and have skewed impact factors [22]. The same would apply for other subspecialties such as perioperative medicine. With both basic science pain and clinical pain management occupying a large part of the database, they may have prevented other subspecialties areas from being highlighted. However, the framework of this study allows articles within each area to be represented and highlighted.

Ramsdell and associates compared the impact factors of pediatrics versus pain on anesthesiologist and presented the low impact of pediatric articles [22]. This study supports this with pediatrics and obstetrics representing the smallest percentiles of articles (both 3.4%). Furthermore, the most-cited pediatric article ranked only #53 overall and the most-cited obstetric articled ranked #222 overall. This is in contrast to the least most-cited chronic and basic science pain articles still ranking highly at #73 and #100 respectively. This clearly exemplifies the difference in impact factors for obstetrics and pediatric anesthesiologist specialty articles as compared to anesthesiologist pain studies. While this could be due to differences in the amount of pediatric anesthesiologist clinical trials or related basic science compared to pain research, other differences in emphasis in studies could also be a factor. One, in particular, is the predominance of studies related to issues in obtaining consent that are emphasized within pediatric anesthesiology and may not be as relevant and, therefore, cited as commonly by non-pediatric anesthesiologist studies. This could also apply to obstetrics patients and its associated difficulties in research. The decision to exclude articles pertaining to basic science research and non-human studies could also have contributed to this bias as many obstetric studies are performed on animals due to ethical concerns of performing a similar study on humans. The lack of research published in these areas could be an area of interest to future researchers looking to develop a niche.

Certainly the articles presented likely have made an impact on the literature of anesthesiology as all articles have been cited at least 100 times. This is especially relevant when 46% of all published clinical articles are never cited [11]. One flaw of citation classics, however, is that they are based on the assumption that authors are appropriately citing especially relevant and important studies. This may not also be the case. Authors are obviously most familiar with their own or work of their colleagues. These networks of authors and their work may lead to self-propagation in a given area [24,25]. Bornmann and Daniel proposed "non-scientific" factors that lead to authors citing other works, including time-dependent, field-dependent, journal-dependent, article-dependent, and author/reader-dependent factors [26]. It is not clear as to how these social networks affect the growth of the anesthesiologist knowledge base or its overall impression in the medical community. This would be a future area of investigation.

While this article presents an update of earlier citation classics in anesthesiologist, most of the articles featured are still almost 20 years old. The year with the most publications in our database was 1986. This is not unexpected, as it has been reported that the true impact of an article cannot be assessed until 20 years after its publication with an article's maximum citations per year occurring three to 10 years after publication. This time period varies with specialty as different journals and areas have different citation half-lives [27]. Finally, after an article's highest number of citations peak in a given year, it will eventually be incorporated into common knowledge and no longer be as frequently cited, or its relevance will wane with new data supplanting or augmenting it. Thus, many important articles are lost to time [15].

The year of publication also affects an article's citations because electronic citation records were initially developed in 1979. Therefore, there is a bias to cite articles published since 1981 [12]. This presents two issues that this paper was unable to resolve. First, the articles retrieved by our search that were published prior to 1979 likely represent especially influential papers as they are not routinely digitized and therefore require manual retrieval in order to be cited within the era of electronic submissions. Moreover, there is a retrieval bias in this study that may have missed some highly-cited articles that were published prior to 1979 as these studies would not populate an electronic bibliometric search.

There are other limitations within our study that should be recognized. First, by using a broad search strategy, this study retrieved almost 19,500 articles that required manual review by the authors (RT, AR) in order to remove articles not primarily focusing on anesthesiologist topics. Using predetermined criteria for manual review of the preliminary database, it is unlikely that personal bias impacted the final data. In addition, the lists were reviewed individually and then the results were compared to minimize a single author influencing the results. Finally, the final lists were reviewed with many experts in the field who agreed with their validity as landmark or classic articles. Another limitation of this paper is the selection of only anesthesiologist journals published in English that would have failed to capture landmark articles published in other languages. Language barriers are a known bias to citation classics as authors are more likely to cite articles in their own language and English articles are more likely to be cited overall [4,9,10,28]. While the United Kingdom and United States of America have historically contributed to over half the anesthesia literature [18], many countries are starting to look at their researchers' contributions to individual fields as well as the international fund of knowledge [29]. Representation of research in the anesthesia literature by groups from other countries has changed over the previous decade; Although the Unites States continues to publish more articles than any country, the percent of articles from the United States and United Kingdom have decreased and other countries such as Turkey, China, and India have contributed an increasing the percentage of manuscripts to the anesthesia literature [30]. With the change in health care in the United States and the evolving global economy, it will be interesting to see how future citation classics compare to current data with respect to the sources of literature.

## Conclusions

In summary, since the advent of anesthesiology and especially over the previous 60 years, the body of knowledge in anesthesiology has flourished. We provide a review of landmark papers in anesthesiology. This review could be used for practioners of all levels of training. Residents and junior attendings could use this article as a reference to articles with historical interest. Senior attendings and administrators could use this article to see the citation counts of their works compared to colleagues for academic interest. To all clinicians, classic articles within the various anesthesiology specialties are especially relevant to affect patient care, future research and as sources of inspiration to the academic pursuit of the field.

## Abbreviations

JCR: Journal Citation Report; SCI Expanded: Science Citation Expanded.

## Competing interests

The authors declare that they have no competing interests.

## Authors' contributions

RT conceived of the study and wrote the manuscript. JB participated in writing and editing the manuscript. TP participated in writing and editing the manuscript. AR conceived of the study and wrote the manuscript.

## Pre-publication history

The pre-publication history for this paper can be accessed here:

http://www.biomedcentral.com/1471-2253/11/24/prepub

## Supplementary Material

Additional file 1 **Most cited articles in anesthesiology by subspecialty**. Click here for file
