# Supplementary material for: A bibliometric search of citation classics in anesthesiology
Source: BMC Anesthesiol. 2011 Dec 12;11:24. doi: 10.1186/1471-2253-11-24 (PMC3261113; doi:10.1186/1471-2253-11-24)
Supplement: Additional file 1 — Most cited articles in anesthesiology by subspecialty. [file 1471-2253-11-24-S1.DOC]

Additional Files: Most cited articles in anesthesiology by subspecialty.

| **Specialty Rank. (Overall Rank). First author. Title. Journal Year. (Times Cited)** |
| --- |
| **AIRWAY**   1. (19) Cormack RS. Difficult tracheal intubation in obstetrics. Anaesthesia 1984. (793) 2. (23) Mendelson CL. The aspiration of stomach contents into the lungs during obstetric anesthesia. Am J Obstet Gynecol 1946. (702) 3. (26) Mallampati SR. A clinical sign to predict difficult tracheal intubation - a prospective-study. Can Anaesth Soc J 1985. (681) 4. (77) Samsoon GLT. Difficult tracheal intubation - a retrospective study. Anaesthesia 1987. (437) 5. (84) Benumof JL. Management of the difficult adult airway - with special emphasis on awake tracheal intubation. Anesthesiology 1991. (424) 6. (96) Sellick BA. Cricoid pressure to control regurgitation of stomach contents during induction of anaesthesia. Lancet 1961. (398) 7. (117) Brain AIJ. The laryngeal mask - a new concept in airway management. Brit J Anaesth 1983. (366) 8. (135) Roberts RB. Reducing risk of acid aspiration during cesarean-section. Anesth Analg 1974. (344) 9. (151) Caplan RA. Practice guidelines for management of the difficult airway - an updated report by the American Society of Anesthesiologists task force on management of the difficult airway. Anesthesiology 2003. (329) 10. (294) Wilson ME. Predicting difficult intubation. Brit J Anaesth 1988. (243) 11. (333) Olsson GL. Aspiration during anesthesia - a computer-aided study of 185-358 anesthetics. Acta Anaesth Scand 1986. (227) 12. (359) Benumof JL. Laryngeal mask airway and the ASA difficult airway algorithm. Anesthesiology 1996. (217) 13. (340) Warner MA. Clinical significance of pulmonary aspiration during the perioperative period. Anesthesiology 1993. (202) 14. (476) King BD. Reflex circulatory responses to direct laryngoscopy and tracheal intubation performed during general anesthesia. Anesthesiology 1951. (195) 15. (490) Henderson JJ. Difficult airway society guidelines for management of the unanticipated difficult intubation. Anaesthesia 2004. (192) 16. (503) Stoelting RK. Circulatory changes during direct laryngoscopy and tracheal intubation - influence of duration of laryngoscopy with or without prior lidocaine. Anesthesiology 1977. (191) 17. (532) Crosby ET. The unanticipated difficult airway with recommendations for management. Can J Anaesth 1998. (186) 18. (621) Verghese C. Survey of laryngeal mask airway usage in 11,910 patients: safety and efficacy for conventional and nonconventional usage. Anesth Analg 1996. (176) 19. (627) Cooper JD. Evolution of tracheal injury due to ventilatory assistance through cuffed tubes - a pathologic study. Ann Surg 1969. (176) 20. (652) Brain AIJ. The intubating laryngeal mask. II: a preliminary clinical report of a new means of intubating the trachea. Brit J Anaesth 1997. (173) |
| **CARDIOTHORACIC AND VASCULAR ANESTHESIOLOGY**   1. (15) Roach GW. Adverse cerebral outcomes after coronary bypass surgery. New Engl J Med 1996. (843) 2. (33) Mangano DT. Perioperative cardiac morbidity. Anesthesiology 1990. (651) 3. (49) Harker LA. Mechanism of abnormal bleeding in patients undergoing cardiopulmonary bypass - acquired transient platelet dysfunction associated with selective alpha-granule release. Blood 1980. (#49 / 535) 4. (48) Butler J. Inflammatory response to cardiopulmonary bypass. Ann Thorac Surg 1993. (469) 5. (62) Royston D. Effect of aprotinin on need for blood-transfusion after repeat open-heart-surgery. Lancet 1987. (458) 6. (85) Slogoff S. Does perioperative myocardial ischemia lead to postoperative myocardial-infarction. Anesthesiology 1985. (424) 7. (95) Bidstrup BP. Reduction in blood loss and blood use after cardiopulmonary bypass with high dose aprotinin (trasylol). J Thorac Cardiov Sur 1989. (398) 8. (121) Woodman RC. Bleeding complications associated with cardiopulmonary bypass. Blood 1990. (360) 9. (122) Rao TLK. Reinfarction following anesthesia in patients with myocardial infarction. Anesthesiology 1983. (354) 10. (129) Mangano DT. The risk associated with aprotinin in cardiac surgery. New Engl J Med 2006. (350) 11. (132) Furnary AP. Continuous intravenous insulin infusion reduces the incidence of deep sternal wound infection in diabetic patients after cardiac surgical procedures. Ann Thorac Surg 1999. (348) 12. (133) Adams JE. Diagnosis of perioperative myocardial infarction with measurement of cardiac troponin I. New Engl J Med 1994. (347) 13. (150) Wan S. Inflammatory response to cardiopulmonary bypass - mechanisms involved and possible therapeutic strategies. Chest 1997. (330) 14. (192) Gardner TJ. Stroke following coronary artery bypass grafting: a 10-year study. Ann Thorac Surg 1985. (298) 15. (228) Flacke JW. Reduced narcotic requirement by clonidine with improved hemodynamic and adrenergic stability in patients undergoing coronary bypass surgery. Anesthesiology 1987. (276) 16. (231) Svensson LG. Deep hypothermia with circulatory arrest. Determinants of stroke and early mortality in 656 patients. J Thorac Cardiov Surg 1993. (274) 17. (240) Zhao ZQ. Inhibition of myocardial injury by ischemic postconditioning during reperfusion: comparison with ischemic preconditioning. Am J Physiol Heart Circ Physiol 2003. (270) 18. (244) Furnary AP. Continuous insulin infusion reduces mortality in patients with diabetes undergoing coronary artery bypass grafting. J Thorac Cardiov Sur 2003. (268) 19. (290) Cremer J. Systemic inflammatory response syndrome after cardiac operations. Ann Thorac Surg 1996. (244) 20. (295) Nussmeier NA. Neuropsychiatric complications after cardiopulmonary bypass - cerebral protection by a barbiturate. Anesthesiology 1986. (243) |
| **GENERAL ANESTHESIOLOGY AND PHYSIOLOGY**   1. (28) Kurz A. Perioperative normothermia to reduce the incidence of surgical-wound infection and shorten hospitalization. New Engl J Med 1996. (675) 2. (41) Owens WD. ASA physical status classifications - study of consistency of ratings. Anesthesiology 1978. (569) 3. (136) Cooper JB. Preventable anesthesia mishaps:a study of human factors. Anesthesiology 1978. (342) 4. (138) Frank SM. Perioperative maintenance of normothermia reduces the incidence of morbid cardiac events. A randomized clinical trial. Jama 1997. (341) 5. (143) Cooper JB. An analysis of major errors and equipment failures in anesthesia management - considerations for prevention and detection. Anesthesiology 1984. (340) 6. (206) Gross JB. Practice guidelines for sedation and analgesia by non-anesthesiologists - an updated report by the American Society of Anesthesiologists task force on sedation and analgesia by non-anesthesiologists. Anesthesiology 2002. (288) 7. (211) Schmied H. Mild hypothermia increases blood loss and transfusion requirements during total hip arthroplasty. Lancet 1996. (286) 8. (212) Prys-Roberts C. Studies of anaesthesia in relation to hypertension. II. Haemodynamic consequences of induction and endotracheal intubation. Brit J Anaesth 1971. (285) 9. (213) Price HL. Sympatho-adrenal responses to general anesthesia in man and their relation to hemodynamics. Anesthesiology 1959. (285) 10. (233) Denborough MA. Anaesthetic deaths in a family. Lancet 1960. (274) 11. (252)Weissman C. The metabolic response to stress - an overview and update. Anesthesiology 1990. (264) 12. (296) Keats AS. ASA classification of physical status - recapitulation. Anesthesiology 1978. (243) 13. (360) Joris JL. Hemodynamic changes during laparoscopic cholecystectomy. Anesth Analg 1993. (217) 14. (411) Tiret L. Complications associated with anesthesia - a prospective survey in France. Can Anaesth Soc J 1986. (206) 15. (413) Prys-Roberts C. Studies of anaesthesia in relation to hypertension. I. Cardiovascular responses of treated and untreated patients. Brit J Anaesth 1971. (206) 16. (428) Sessler DI. Mild perioperative hypothermia. New Engl J Med 1997. (202) 17. (443) Rubinstein EH. Skin-surface temperature gradients correlate with fingertip blood flow in humans. Anesthesiology 1990. (201) 18. (473) Bruce DL. Causes of death among anesthesiologists: a 20-year survey. Anesthesiology 1968. (195) 19. (474) Eger EI. Hazards of nitrous oxide anesthesia in bowel obstruction and pneumothorax. Anesthesiology 1965. (195) 20. (483) Goldman L. Risks of general anesthesia and elective operation in the hypertensive patient. Anesthesiology 1979. (194) |
| **HEAD AND NECK SURGERY**   1. (10) Lassen NA. Cerebral blood flow and oxygen consumption in man. Physiol Rev 1959. (1015) 2. (25) Paulson OB. Cerebral autoregulation. Cerebrovas Brain Met 1990. (697) 3. (47) Reivich M. Arterial pCO2 and cerebral hemodynamics. Am J Physiol 1964. (539) 4. (70) Rampil IJ. A primer for EEG signal processing in anesthesia. Anesthesiology 1998. (443) 5. (71) Rosner MJ. Cerebral perfusion pressure: management protocol and clinical results. J Neurosurg 1995. (442) 6. (74) Muizelaar JP. Adverse effects of prolonged hyperventilation in patients with severe head injury: a randomized clinical trial. J Neurosurg 1991. (439) 7. (107) Thomas DJ. Effect of haematocrit on cerebral blood-flow in man. Lancet 1977. (377) 8. (123) Rosomoff HL. Cerebral blood flow and cerebral oxygen consumption during hypothermia. Am J Physiol 1954. (354) 9. (127) Scott JC. EEG quantitation of narcotic effect: the comparative pharmacodynamics of fentanyl and alfentanil. Anesthesiology 1985. (352) 10. (153) Weir B. Time course of vasospasm in man. J Neurosurg 1978. (329) 11. (173) Giller CA. Cerebral arterial diameters during changes in blood pressure and carbon dioxide during craniotomy. Neurosurgery 1993. (313) 12. (187) Smith AL. Cerebral blood flow and metabolism: effects of anesthetic drugs and techniques. Anesthesiology 1972. (302) 13. (188) Cohen PJ. Effects of hypoxia and normocarbia on cerebral blood flow and metabolism in conscious man. J Appl Physiol 1967. (302) 14. (189) Shiozaki T. Effect of mild hypothermia on uncontrollable intracranial hypertension after severe head injury. J Neurosurg 1993. (301) 15. (191) Clifton GL. A phase II study of moderate hypothermia in severe brain injury. J Neurotraum 1993. (298) 16. (194) Marion DW. The use of moderate therapeutic hypothermia for patients with severe head injuries: a preliminary report. J Neurosurg 1993. (295) 17. (200) Pierce EC. Cerebral circulation and metabolism during thiopental anesthesia and hyperventilation in man. J Clin Invest 1962. (291) 18. (237) Grubb RL. Effects of subarachnoid hemorrhage on cerebral blood volume, blood flow, and oxygen utilization in humans. J Neurosurg 1977. (272) 19. (276) Clark DL. Neurophysiologic effects of general anesthetics. I. Electroencephalogram and sensory evoked responses in man. Anesthesiology 1973. (251) 20. (289) Sundt TM. Cerebral blood flow measurements and electroencephalograms during carotid endarterectomy. J Neurosurg 1974. (245) |
| **MONITORS**   1. (42) Glass PS. Bispectral analysis measures sedation and memory effects of propofol, midazolam, isoflurane, and alfentanil in healthy volunteers. Anesthesiology 1997. (568) 2. (92) Daniel WG. Safety of transesophageal echocardiography. A multicenter survey of 10,419 examinations. Circulation 1991. (404) 3. (131) Sandham JD. A randomized, controlled trial of the use of pulmonary-artery catheters in high-risk surgical patients. New Engl J Med 2003. (348) 4. (198) Pauca AL. Prospective evaluation of a method for estimating ascending aortic pressure from the radial artery pressure waveform. Hypertension 2001. (291) 5. (225) Shanewise JS. ASE/SCA guidelines for performing a comprehensive intraoperative multiplane transesophageal echocardiography examination: recommendations of the American Society of Echocardiography Council for Intraoperative Echocardiography and the Society of Cardiovascular Anesthesiologists task force for certification in perioperative transesophageal echocardiography. Anesth Analg; J Am Soc Echocardiog 1999. (184+95) 6. (261) Gan TJ. Bispectral index monitoring allows faster emergence and improved recovery from propofol, alfentanil, and nitrous oxide anesthesia. Anesthesiology 1997. (260) 7. (263) Gardner RM. Direct blood pressure measurement – dynamic response requirements. Anesthesiology 1981. (260) 8. (307) Thys DM. Practice guidelines for perioperative transesophageal echocardiography - a report by the American Society of Anesthesiologists and the Society of Cardiovascular Anesthesiologists task force on transesophageal echocardiography. Anesthesiology 1996. (238) 9. (334) Bedford RF. Complications of percutaneous radial-artery cannulation: an objective prospective study in man. Anesthesiology 1973. (227) 10. (342) Sebelz PS. A multicenter study of bispectral electroencephalogram analysis for monitoring anesthetic effect. Anesth Analg 1997. (224) 11. (366) Myles PS. Bispectral index monitoring to prevent awareness during anaesthesia: the B-AWARE randomised controlled trial. Lancet 2004. (214) 12. (401) Liu J. Electroencephalographic bispectral index correlates with intraoperative recall and depth of propofol-induced sedation. Anesth Analg 1997. (207) 13. (424) Randolph AG. Ultrasound guidance for placement of central venous catheters: a meta-analysis of the literature. Crit Care Med 1996. (203) 14. (467) Johansen JW. Development and clinical application of electroencephalographic bispectrum monitoring. Anesthesiology 2000. (195) 15. (468) Song DJ. Titration of volatile anesthetics using bispectral index facilitates recovery after ambulatory anesthesia. Anesthesiology 1997. (195) 16. (482) Severinghaus JW. Accuracy of response of 6 pulse oximeters to profound hypoxia. Anesthesiology 1987. (194) 17. (499) Smith WD. Measuring the performance of anesthetic depth indicators. Anesthesiology 1996. (191) 18. (599) Katoh T. Electroencephalographic derivatives as a tool for predicting the depth of sedation and anesthesia induced by sevoflurane. Anesthesiology 1998. (179) 19. (641) Tavernier B. Systolic pressure variation as a guide to fluid therapy in patients with sepsis-induced hypotension. Anesthesiology 1998. (174) 20. (647) Shah KB. A review of pulmonary artery catheterization in 6,245 patients. Anesthesiology 1984. (174) |
| **OBSTETRICAL ANESTHESIOLOGY**   1. (222) Cohen EN. Anesthesia, pregnancy, and miscarriage: a study of operating room nurses and anesthetists. Anesthesiology 1971. (281) 2. (259) Thorp JA. The effect of intrapartum epidural analgesia on nulliparous labor: a randomized, controlled, prospective trial. Am J Obstet Gynecol 1993. (261) 3. (277) Hawkins JL. Anesthesia-related deaths during obstetric delivery in the United States, 1979-1990. Anesthesiology 1997. (250) 4. (299) Scanlon JW. Neurobehavioral responses of newborn infants after maternal epidural anesthesia. Anesthesiology 1974. (242) 5. (320) Kaunitz AM. Causes of maternal mortality in the United States. Obstet Gynecol 1985. (234) 6. (337) Knilljon RP. Anesthetic practice and pregnancy. Controlled survey of women anesthetists in United Kingdom. Lancet 1972. (226) 7. (446) Pritchar JA. Changes in blood volume during pregnancy and delivery. Anesthesiology 1965. (201) 8. (477) Polley LS. Relative analgesic potencies of ropivacaine and bupivacaine for epidural analgesia in labor: implications for therapeutic indexes. Anesthesiology 1999. (194) 9. (494) Kennell, J. Continuous emotional support during labor in a US hospital. A randomized controlled trial. JAMA 1991. (192) 10. (542) Lester BM. Regional obstetric anesthesia and newborn behavior: a reanalysis toward synergistic effects. Child Dev 1982. (186) 11. (602) Nimmo WS. Narcotic analgesics and delayed gastric emptying during labor. Lancet 1975. (179) 12. (609) Rochat RW. Maternal mortality in the United States: report from the Maternal Mortality Collaborative. Obstet Gynecol 1988. (178) 13. (767) Ramin SM. Randomized trial of epidural versus intravenous analgesia during labor. Obstet Gynecol 1995. (163) 14. (768) Scott DB. Serious nonfatal complications associated with extradural block in obstetric practice. Brit J Anaesth 1990. (163) 15. (774) Amieltison C. A new neurologic and adaptive capacity scoring system for evaluating obstetric medications in full-term newborns. Anesthesiology 1982. (163) 16. (789) Corbett TH. Birth defects among children of nurse-anesthetists. Anesthesiology 1974. (162) 17. (986) Morgan M. Amniotic fluid embolism. Anaesthesia 1979. (148) 18. (1053) Collis RE. Randomized comparison of combined spinal-epidural and standard epidural analgesia in labor. Lancet 1995. (143) 19. (1072) Norris MC. Complications of labor analgesia: epidural versus combined spinal epidural techniques. Anesth Analg 1994. (142) 20. (1117) Eisenach JC. Patient-controlled analgesia following cesarean section: a comparison with epidural and intramuscular narcotics. Anesthesiology 1988. (139) |
| **PAIN, ACUTE AND BASIC**   1. (1) Zimmermann M. Ethical guidelines for investigations of experimental pain in conscious animals. Pain 1983. (2729) 2. (3) Martin WR. Effects of morphine-like and nalorphine-like drugs in nondependent and morphine-dependent chronic spinal dog. J Pharmacol Exp Ther 1976. (2461) 3. (4) Bennett GJ. A peripheral mononeuropathy in rat that produces disorders of pain sensation like those seen in man. Pain 1988. (1891) 4. (7) Coderre TJ. Contribution of central neuroplasticity to pathological pain: review of clinical and experimental evidence. Pain 1993. (1183) 5. (9) Woolf CJ. The induction and maintenance of central sensitization is dependent on N-methyl-D-aspartic acid receptor activation; implications for the treatment of post-injury pain hypersensitivity states. Pain 1991. (1059) 6. (11) Hylden JLK. Intrathecal morphine in mice: a new technique. Eur J Pharmacol 1980. (990) 7. (16) Vonvoigtlander PF. U-50,488: a selective and structurally novel non-Mu (kappa) opioid agonist. J Pharmacol Exp Ther 1983. (839) 8. (35) Meller ST. Nitric oxide (NO) and nociceptive processing in the spinal cord. Pain 1993. (634) 9. (37) Yaksh TL. Intrathecal morphine inhibits substance P release from mammalian spinal cord in vivo. Nature 1980. (624) 10. (38) Mayer DJ. Central nervous system mechanisms of analgesia. Pain 1976. (620) 11. (44) Yaksh TL. Narcotic analgestics: CNS sites and mechanisms of action as revealed by intracerebral injection techniques. Pain 1978. (558) 12. (45) Sindrup SH. Efficacy of pharmacological treatments of neuropathic pain: an update and effect related to mechanism of drug action. Pain 1999. (545) 13. (51) Yaksh TL. Studies on direct spinal action of narcotics in production of analgesia in rat. J Pharmacol Exp Ther 1977. (532) 14. (64) Fitzgerald M. Capsaicin and sensory neurones - a review. Pain 1983. (454) 15. (82) Gracely RH. Painful neuropathy; altered central processing maintained dynamically by peripheral input. Pain 1992. (428) 16. (87) Lebars D. Diffuse noxious inhibitory controls (DNIC). II. Lack of effect on non-convergent neurons, supraspinal involvement and theoretical implications. Pain 1979. (418) 17. (91) Mense S. Nociception from skeletal muscle in relation to clinical muscle pain. Pain 1993. (405) 18. (98) Mao JR. Mechanisms of hyperalgesia and morphine tolerance: a current view of their possible interactions. Pain 1995. (393) 19. (99) Handwerker HO. Segmental and supraspinal actions on dorsal horn neurons responding to noxious and non-noxious skin stimuli. Pain 1975. (393) 20. (100) Yaksh TL. Behavioral and autonomic correlates of the tactile evoked allodynia produced by spinal glycine inhibition: effects of modulatory receptor systems and excitatory amino acid antagonists. Pain 1989. (390) |
| **PAIN, CHRONIC MANAGEMENT**   1. (2) Melzack R. The McGill Pain Questionnaire: major properties and scoring methods. Pain 1975. (2699) 2. (6) Scott J. Graphic representation of pain. Pain 1976. (1233) 3. (8) Melzack R. The short-form McGill Pain Questionnaire. Pain 1987. (1088) 4. (12) Rosenstiel AK. The use of coping strategies in chronic low back pain patients: relationship to patient characteristics and current adjustment. Pain 1983. (930) 5. (13) Price DD. The validation of visual analog scales as ratio scale measures for chronic and experimental pain. Pain 1983. (911) 6. (14) Kerns RD. The West Haven-Yale Multidimensional Pain Inventory (WHYMPI). Pain 1985. (849) 7. (17) Jensen MP. The measurement of clinical pain intensity: a comparison of six methods. Pain 1986. (821) 8. (24) Revill SI. Reliability of a linear analog for evaluating pain. Anaesthesia 1976. (699) 9. (30) Waddell G. A Fear-Avoidance Beliefs Questionnaire (FABQ) and the role of fear-avoidance beliefs in chronic low back pain and disability. Pain 1993. (665) 10. (34) Vonkorff M. Grading the severity of chronic pain. Pain 1992. (650) 11. (36) Vlaeyen JWS. Fear-avoidance and its consequences in chronic musculoskeletal pain: a state of the art. Pain 2000. (632) 12. (39) Arner S. Lack of analgesic effect of opioids on neuropathic and idiopathic forms of pain. Pain 1988. (584) 13. (40) Farrar JT. Clinical importance of changes in chronic pain intensity measured on an 11-point numerical pain rating scale. Pain 2001. (578) 14. (55) Unruh AM. Gender variations in clinical pain experience. Pain 1996. (482) 15. (56) Flor H. Efficacy of multidisciplinary pain treatment centers: a meta-analytic review. Pain 1992. (472) 16. (63) Chapman CR. Pain measurement: an overview. Pain 1985. (454) 17. (66) Levine JD. Mechanism of placebo analgesia. Lancet 1978. (446) 18. (67) Vlaeyen JWS. Fear of movement/(re)injury in chronic low back pain and its relation to behavioral performance. Pain 1995. (445) 19. (72) Mcquay HJ. Systematic review of antidepressants in neuropathic pain. Pain 1996. (441) 20. (73) Melzack R. Language of pain. Anesthesiology 1971. (440) |
| **PEDIATRIC ANESTHESIOLOGY**   1. (53) Lou HC. Impaired autoregulation of cerebral blood flow in the distressed newborn infant. J Pediatr 1979. (513) 2. (104) Kauffman RE. Guidelines for monitoring and management of pediatric-patients during and after sedation for diagnostic and therapeutic procedures. Pediatrics 1992. (381) 3. (139) Anand KJS. Halothane-morphine compared with high-dose sufentanil for anesthesia and postoperative analgesia in neonatal cardiac surgery. New Engl J Med 1992. (341) 4. (142) Grunau RVE. Pain expression in neonates: facial action and cry. Pain 1987. (340) 5. (152) Marsh B. Pharmacokinetic model driven infusion of propofol in children. Brit J Anaesth 1991. (329) 6. (186) Bieri D. The Faces Pain Scale for the self-assessment of the severity of pain experienced by children: development, initial validation, and preliminary investigation for ratio scale properties. Pain 1990. (302) 7. (346) Perquin CW. Pain in children and adolescents: a common experience. Pain 2000. (223) 8. (387) Craig KD. Pain in the preterm neonate - behavioral and physiological indexes. Pain 1993. (210) 9. (425) Stevens B. Premature infant pain profile: development and initial validation. Clin J Pain 1996. (203) 10. (480) Goodman JE. The epidemiology of pain in children and adolescents: a review. Pain 1991. (194) 11. (501) Varni JW. The Varni/Thompson Pediatric Pain Questionnaire. I. Chronic musculoskeletal pain in juvenile rheumatoid arthritis. Pain 1987. (191) 12. (536) Cohen MM. Pediatric anesthesia morbidity and mortality in the perioperative period. Anesth Analg 1990. (186) 13. (538) Fitzgerald M. Cutaneous hypersensitivity following peripheral tissue damage in newborn infants and its reversal with topical anesthesia. Pain 1989. (186) 14. (557) Lerman J. The pharmacology of sevoflurane in infants and children. Anesthesiology 1994. (184) 15. (568) Mather L. The incidence of postoperative pain in children. Pain 1983. (183) 16. (778) Mcdonald LH. Prolonged nasotracheal intubation: a review of its development in a paediatric hospital. Brit J Anaesth 1965. (163) 17. (882) Anand KJS. Randomized trial of fentanyl anesthesia in preterm babies undergoing surgery: effects on the stress response. Lancet 1987. (155) 18. (884) Greenough A. Pancuronium prevents pneumothoraces in ventilated premature babies who actively expire against positive pressure inflation. Lancet 1984. (155) 19. (914) Steward DJ. Preterm infants are more prone to complications following minor surgery than are term infants. Anesthesiology 1982. (153) 20. (940) Hicks CL. The Faces Pain Scale-Revised: toward a common metric in pediatric pain measurement. Pain 2001. (150) |
| **PREOPERATIVE MANAGEMENT**   1. (79) Eagle KA. ACCAHA guideline update for perioperative cardiovascular evaluation for noncardiac surgery - executive summary. A report of the American College of Cardiology/American Heart Association task force on practice guidelines (committee to update the 1996 guidelines on perioperative cardiovascular evaluation for noncardiac surgery). Circulation; Anesth Analg 2002. (323+110) 2. (197) Gokce N. Risk stratification for postoperative cardiovascular events via noninvasive assessment of endothelial function: a prospective study. Circulation 2002. (291) 3. (234) Wallace A. Prophylactic atenolol reduces postoperative myocardial ischemia. Anesthesiology 1998. (272) 4. (255) Chertow GM. Preoperative renal risk stratification. Circulation 1997. (262) 5. (301) Eagle KA. Guidelines for perioperative cardiovascular evaluation for noncardiac surgery. Report of the American College of Cardiology American Heart Association task force on practice guidelines (committee on perioperative cardiovascular evaluation for noncardiac surgery). Circulation 1996. (241) 6. (306) Boersma E. Predictors of cardiac events after major vascular surgery: role of clinical characteristics, dobutamine echocardiography, and beta-blocker therapy. Jama-J Am Med Assoc 2001. (238) 7. (314) Eagle KA. ACC/AHA guideline update for perioperative cardiovascular evaluation for noncardiac surgery - executive summary: a report of the American College of Cardiology/American Heart Association Task Force on Practice Guidelines (Committee to Update the 1996 Guidelines on Perioperative Cardiovascular Evaluation for Noncardiac Surgery). J Am Coll Cardiol 2002. (235) 8. (364) Poldermans D. Dobutamine stress echocardiography for assessment of perioperative cardiac risk in patients undergoing major vascular surgery. Circulation 1993. (215) 9. (400) Poldermans D. Statins are associated with a reduced incidence of perioperative mortality in patients undergoing major noncardiac vascular surgery. Circulation 2003. (207) 10. (422) Lindenauer PK. Perioperative beta-blocker therapy and mortality after major noncardiac surgery. New Engl J Med 2005. (203) 11. (432) Foster ED. Risk of noncardiac operation in patients with defined coronary disease: The Coronary Artery Surgery Study (CASS) registry experience. Ann Thorac Surg 1986. (202) 12. (486) Eagle, KA. Guidelines for perioperative cardiovascular evaluation for noncardiac surgery. J Am Coll Cardiol 1996. (193) 13. (560) Berlauk JF. Preoperative optimization of cardiovascular hemodynamics improves outcome in peripheral vascular surgery. A prospective, randomized clinical trial. Ann Surg 1991. (184) 14. (706) Goldman L. Cardiac risks and complications of noncardiac surgery. Ann Intern Med 1983. (169) 15. (726) Durazzo AES. Reduction in cardiovascular events after vascular surgery with atorvastatin: a randomized trial. J Vasc Surg 2004. (166) 16. (727) Auerbach AD. Beta-blockers and reduction of cardiac events in noncardiac surgery - scientific review. JAMA 2002. (166) 17. (751) Eagle KA. Cardiac risk of noncardiac surgery: influence of coronary disease and type of surgery in 3368 operations. CASS Investigators and University of Michigan Heart Care Program. Coronary Artery Surgery Study. Circulation 1997. (164) 18. (861) Raby KE. The effect of heart rate control on myocardial ischemia among high-risk patients after vascular surgery. Anesth Analg 1999. (156) 19. (902) Devereaux PJ. How strong is the evidence for the use of perioperative beta blockers in non-cardiac surgery? Systematic review and meta-analysis of randomised controlled trials. Brit Med J 2005. (153) 20. (991) Devereaux PJ. Effects of extended-release metoprolol succinate inpatients undergoing non-cardiac surgery (POISE trial): a randomised controlled trial. Lancet 2008. (147) |
| **POSTOPERATIVE CARE**   1. (22) Watcha MF. Postoperative nausea and vomiting. Its etiology, treatment, and prevention. Anesthesiology 1992. (713) 2. (31) Woolf CJ. Preemptive analgesia treating postoperative pain by preventing the establishment of central sensitization. Anesth Analg 1993. (654) 3. (57) Aldrete JA. Postanesthetic recovery score. Anesth Anal Curr Res 1970. (470) 4. (119) Kehlet H. The value of multimodal or balanced analgesia in postoperative pain treatment. Anesth Analg 1993. (365) 5. (145) Wall PD. The prevention of postoperative pain. Pain 1988. (336) 6. (238) Katz J. Preemptive analgesia. Clinical evidence of neuroplasticity contributing to postoperative pain. Anesthesiology 1992. (271) 7. (239) Cohen FL. Postsurgical pain relief: patients’ status and nurses’ medication choices. Pain 1980. (271) 8. (243) Kehlet H. Multimodal approach to control postoperative pathophysiology and rehabilitation. Brit J Anaesth 1997. (269) 9. (248) Apfel CC. A simplified risk score for predicting postoperative nausea and vomiting: conclusions from cross-validations between two centers. Anesthesiology 1999. (264) 10. (374) Palazzo MG. Anesthesia and emesis. I. Etiology. Can Anaesth Soc J 1984. (213) 11. (376) Gan TJ. Consensus guidelines for managing postoperative nausea and vomiting. Anesth Analg 2003. (#376 / 212) 12. (379) Kehlet H. Anaesthesia, surgery, and challenges in postoperative recovery. Lancet 2003. (#379 / 211) 13. (457) Richmond CE. Preoperative morphine preempts postoperative pain. Lancet 1993. (197) 14. (458) Dahl JB. The value of preemptive analgesia in the treatment of postoperative pain. Brit J Anaesth 1993. (197) 15. (504) Steward DJ. Simplified scoring system for postoperative recovery room. Can Anaesth Soc J 1975. (191) 16. (524) Moiniche S. A qualitative and quantitative systematic review of preemptive analgesia for postoperative pain relief: the role of timing of analgesia. Anesthesiology 2002. (187) 17. (525) Koivuranta M. A survey of postoperative nausea and vomiting. Anaesthesia 1997. (187) 18. (534) Aldrete JA. The postanesthesia recovery score revisited. J Clin Anesth 1995. (186) 19. (547) Apfelbaum JL. Postoperative pain experience: results from a national survey suggest postoperative pain continues to be undermanaged. Anesth Analg 2003. (185) 20. (570) Henzi I. Dexamethasone for the prevention of postoperative nausea and vomiting: a quantitative systematic review. Anesth Analg 2000. (182) |
| **PHARMACOLOGY**   1. (20) White PF. Ketamine - its pharmacology and therapeutic uses. Anesthesiology 1982. (787) 2. (27) Kalow W. A method for the detection of atypical forms of human serum cholinesterase; determination of dibucaine numbers. Can J Biochem Phys 1957. (678) 3. (29) Mihic SJ. Sites of alcohol and volatile anaesthetic action on GABA(A) and glycine receptors. Nature 1997. (670) 4. (32) Eger EI. Minimum alveolar anesthetic concentration: a standard of anesthetic potency. Anesthesiology 1965. (653) 5. (48) Gronert GA. Malignant hyperthermia. Anesthesiology 1980. (535) 6. (65) Reves JG. Midazolam: pharmacology and uses. Anesthesiology 1985. (449) 7. (69) Quasha AL. Determination and applications of MAC. Anesthesiology 1980. (444) 8. (80) Maze M. Alpha-2 adrenoceptor agonists: defining the role in clinical anesthesia. Anesthesiology 1991. (433) 9. (106) Maclennan DH. Ryanodine receptor gene is a candidate for predisposition to malignant hyperthermia. Nature 1990. (378) 10. (116) Sebel PS. Propofol: a new intravenous anesthetic. Anesthesiology 1989. (366) 11. (134) Harrington RA. Metoclopramide. An updated review of its pharmacological properties and clinical use. Drugs 1983. (345) 12. (147) Thesleff S. The mode of neuromuscular block caused by acetylcholine, nicotine, decamethonium and succinylcholine. Acta Physiol Scand 1955. (335) 13. (159) Stevens WC. Cardiovascular effects of a new inhalation anesthetic, forane, in human volunteers at constant arterial carbon dioxide tension. Anesthesiology 1971. (325) 14. (164) Ali HH. Monitoring of neuromuscular function. Anesthesiology 1976. (318) 15. (172) Stevens WC. Minimum alveolar concentrations (MAC) of isoflurane with and without nitrous oxide in patients of various ages. Anesthesiology 1975. (314) 16. (174) Eger EI. Cardiovascular effects of halothane in man. Anesthesiology 1970. (310) 17. (176) Gregory GA. Relationship between age and halothane requirement in man. Anesthesiology 1969. (307) 18. (180) Smith I. Propofol. An update on its clinical use. Anesthesiology 1994. (305) 19. (195) Eger EI. Isoflurane: a review. Anesthesiology 1981. (295) 20. (199) Reich DL. Ketamine: an update on the first twenty-five years of clinical experience. Can J Anaesth 1989. (291) |
| **REGIONAL ANESTHESIOLOGY AND LOCAL ANESTHETIC PHARMACOLOGY**   1. (5) Hille B. Local-anesthetics: hydrophilic and hydrophobic pathways for drug-receptor reaction. J Gen Physiol 1977. (1373) 2. (18) Cousins MJ. Intrathecal and epidural administration of opioids. Anesthesiology 1984. (794) 3. (21) Neher E. Local anesthetics transiently block currents through single acetylcholine-receptor channels. J Physiol 1978. (740) 4. (43) Albright GA. Cardiac arrest following regional anesthesia with etidocaine or bupivacaine. Anesthesiology 1979. (559) 5. (46) Wang JK. Pain relief by intrathecally applied morphine in man. Anesthesiology 1979. (541) 6. (50) Yaksh TL. Spinal opiate analgesia: characteristics and principles of action. Pain 1981. (534) 7. (52) Yeager MP. Epidural anesthesia and analgesia in high-risk surgical patients. Anesthesiology 1987. (530) 8. (59) Behar M. Epidural morphine in treatment of pain. Lancet 1979. (469) 9. (68) Butterworth JF. Molecular mechanisms of local anesthesia: a review. Anesthesiology 1990. (444) 10. (86) Rodgers A. Reduction of postoperative mortality and morbidity with epidural or spinal anaesthesia: results from overview of randomised trials. Brit Med J 2000. (420) 11. (93) Liu S. Epidural anesthesia and analgesia. Their role in postoperative outcome. Anesthesiology 1995. (402) 12. (105) Scott DB. Acute toxicity of ropivacaine compared with that of bupivacaine. Anesth Analg 1989. (380) 13. (108) Bromage PR. Epidural narcotics for postoperative analgesia. Anesth Analg 1980. (375) 14. (115) Auroy Y. Serious complications related to regional anesthesia: results of a prospective survey in France. Anesthesiology 1997. (366) 15. (118) Vandermeulen EP. Anticoagulants and spinal-epidural anesthesia. Anesth Analg 1994. (365) 16. (144) Rigler ML. Cauda equina syndrome after continuous spinal anesthesia. Anesth Analg 1991. (339) 17. (146) Ballantyne JC. The comparative effects of postoperative analgesic therapies on pulmonary outcome: cumulative meta-analyses of randomized, controlled trials. Anesth Analg 1998. (335) 18. (155) Tuman KJ. Effects of epidural anesthesia and analgesia on coagulation and outcome after major vascular surgery. Anesth Analg 1991. (328) 19. (165) Knudsen K. Central nervous and cardiovascular effects of i.v. infusions of ropivacaine, bupivacaine and placebo in volunteers. Brit J Anaesth 1997. (317) 20. (170) Tverskoy M. Postoperative pain after inguinal herniorrhaphy with different types of anesthesia. Anesth Analg 1990. (315) |
| **PULMONARY PHYSIOLOGY**   1. (54) Boyd O. A randomized clinical trial of the effect of deliberate perioperative increase of oxygen delivery on mortality in high-risk surgical patients. JAMA 1993. (485) 2. (78) Froese AB. Effects of anesthesia and paralysis on diaphragmatic mechanics in man. Anesthesiology 1974. (435) 3. (128) Qvist J. Hemodynamic responses to mechanical ventilation with PEEP: the effect of hypervolemia. Anesthesiology 1975. (352) 4. (140) Caplan RA. Adverse respiratory events in anesthesia: a closed claims analysis. Anesthesiology 1990. (341) 5. (141) Craig DB. Postoperative recovery of pulmonary function. Anesth Analg 1981. (341) 6. (210) Catley DM. Pronounced, episodic oxygen desaturation in the postoperative period: its association with ventilatory pattern and analgesic regimen. Anesthesiology 1985. (287) 7. (335) Brismar B. Pulmonary densities during anesthesia with muscular relaxation - a proposal of atelectasis. Anesthesiology 1985. (226) 8. (352) Nunn JF. Hypoxaemia after general anaesthesia. Lancet 1962. (221) 9. (363) Marshall BE. Hypoxemia during and after anesthesia. Anesthesiology 1972. (216) 10. (251) Wittgen CM. Analysis of the hemodynamic and ventilatory effects of laparoscopic cholecystectomy. Arch Surg-Chicago 1991. (264) 11. (394) Morgan BC. Hemodynamic effects of intermittent positive pressure respiration. Anesthesiology 1966. (209) 12. (421) Kelman GR. Cardiac output and arterial blood-gas tension during laparoscopy. Brit J Anaesth 1972. (204) 13. (452) Bay J. Factors influencing arterial PO2 during recovery from anaesthesia. Brit J Anaesth 1968. (199) 14. (459) Joris J. Metabolic and respiratory changes after cholecystectomy performed via laparotomy or laparoscopy. Brit J Anaesth 1992. (197) 15. (475) Nunn JF. Factors influencing arterial oxygen tension during anaesthesia with artificial ventilation. Brit J Anaesth 1965. (195) 16. (664) Rothen HU. Re-expansion of atelectasis during general anesthesia: a computed tomography study. Brit J Anaesth 1993. (172) 17. (690) Tokics L. Lung collapse and gas exchange during general anesthesia: effects of spontaneous breathing, muscle paralysis, and positive end-expiratory pressure. Anesthesiology 1987. (170) 18. (833) Alexander JI. The role of airway closure in postoperative hypoxaemia. Brit J Anaesth 1973. (159) 19. (747) Fletcher R. The concept of deadspace with special reference to the single breath test for carbon dioxide. Brit J Anaesth 1981. (165) 20. (966) Hedenstierna G. Functional residual capacity, thoracoabdominal dimensions, and central blood volume during general-anesthesia with muscle paralysis and mechanical ventilation. Anesthesiology 1985. (149) |
| **FLUID MANAGEMENT AND TRANSFUSION MEDICINE**   1. (83) Stehling LC. Practice guidelines for blood component therapy: a report by the American Society of Anesthesiologists Task Force on Blood Component Therapy. Anesthesiology 1996. (425) 2. (126) Busch OR. Blood transfusions and prognosis in colorectal cancer. New Engl J Med 1993. (352) 3. (183) Kang YG. Intraoperative changes in blood coagulation and thrombelastographic monitoring in liver transplantation. Anesth Analg 1985. (304) 4. (215) Mallett SV. Thrombelastography. Brit J Anaesth 1992. (284) 5. (241) van de Watering LMG. Beneficial effects of leukocyte depletion of transfused blood on postoperative complications in patients undergoing cardiac surgery: a randomized clinical trial. Circulation 1998. (270) 6. (247) Bernstein MJ. Perioperative red blood-cell transfusion. JAMA 1988. (265) 7. (249) Heiss MM. Beneficial effect of autologous blood transfusion on infectious complications after colorectal cancer surgery. Lancet 1993. (264) 8. (254) Opelz G. Dominant effect of transfusions on kidney graft survival. Transplantation 1980. (#254 / 264) 9. (272) Boffard KD. Recombinant factor VIIa as adjunctive therapy for bleeding control in severely injured trauma patients: two parallel randomized, placebo-controlled, double-blind clinical trials. J Trauma 2005. (253) 10. (273) Martinowitz U. Recombinant activated factor VII for adjunctive hemorrhage control in trauma. J Trauma 2001. (253) 11. (279) Rohrer MJ. Effect of hypothermia on the coagulation cascade. Crit Care Med 1992. (249) 12. (281) Mythen MG. Perioperative plasma volume expansion reduces the incidence of gut mucosal hypoperfusion during cardiac surgery. Arch Surg 1995. (247) 13. (310) Virgilio RW. Crystalloid vs colloid resuscitation: is one better? A randomized clinical study. Surgery 1979. (237) 14. (321) Sinclair S. Intraoperative intravascular volume optimisation and length of hospital stay after repair of proximal femoral fracture: randomised controlled trial. Brit Med J 1997. (233) 15. (331) Blumberg N. Relation between recurrence of cancer of the colon and blood transfusion. Brit Med J 1985. (228) 16. (345) Friederich PW. Effect of recombinant activated factor VII on perioperative blood loss in patients undergoing retropubic prostatectomy: a double-blind placebo-controlled randomised trial. Lancet 2003. (223) 17. (354) Brandstrup B. Effects of intravenous fluid restriction on postoperative complications: comparison of two perioperative fluid regimens: a randomized assessor-blinded multicenter trial. Ann Surg 2003. (219) 18. (373) Rosen CB. Perioperative blood-transfusion and determinants of survival after liver resection for metastatic colorectal carcinoma. Ann Surg 1992. (213) 19. (382) Murphy P. Infection or suspected infection after hip replacement surgery with autologous or homologous blood transfusions. Transfusion 1991. (211) 20. (391) Shore-Lesserson L. Thromboelastography-guided transfusion algorithm reduces transfusions in complex cardiac surgery. Anesth Analg 1999. (209) |
